# Effect of irrigation frequency and selenium fertilization on the vegetative growth and biomass yield of *Moringa oleifera* and *Moringa peregrina*

**DOI:** 10.1038/s41598-022-26967-5

**Published:** 2022-12-26

**Authors:** Khalid A. Abdoun, Osman A. Altahir, Ahmed A. Alsagan, Mohammed Y. Alsaiady, Ali M. Alshaikhi, Faisal A. Alshamiry, Ahmed A. Al-Haidary

**Affiliations:** 1 Department of Animal Production, College of Food and Agriculture Sciences, King Saud University, P. O. Box 2460, Riyadh, 11451 Saudi Arabia; 2 Biostatistics Department, Naif Arab University for Security Sciences, Riyadh, Saudi Arabia; 3 King Abdulaziz City for Science and Technology, Riyadh, Saudi Arabia; 4 Development and Research Unit, Arabian Agricultural Services Company (ARASCO), Riyadh, 12311 Saudi Arabia

**Keywords:** Plant sciences, Plant stress responses

## Abstract

To maximize the production value of *Moringa* species, there is a need to understand the morphological effect of irrigation frequency and Se fertilizer levels on *Moringa* species' growth and development. Hence, the aim of this trial was to determine the combined effect of irrigation frequency and Selenium (Se) fertilizer levels on the growth of *Moringa oleifera* (*M. oleifera*) and *Moringa peregrina* (*M. peregrina*) in the central region of the Kingdom of Saudi Arabia. A split-plot arrangement was used, where the treatments were arranged in a Completely Randomized Block Design (CRBD) with three replicates, as the study included two plant species (*M. oleifera* and *M. peregrina*), four irrigation frequencies (7, 10, 15, and 20 days), and three Se levels (0.0, 12.5 and 25 mg/L foliar spray). *M. peregrina* showed slow emergence and plant establishment as compared to *M. oleifera*. The results indicated that plant height, leaves and stems fresh weight increased with the increase in Se level, and were highest when the two plant species were irrigated every 10 days which surpassed all other irrigation frequencies. The findings of this research study indicated that the two *Moringa* species, particularly *M. oleifera* could successfully be grown using drip irrigation at a 10-days irrigation frequency.

## Introduction

In dry and arid regions, Moringa is established using regular irrigation for the first 2 months, and once established, Moringa rarely needs watering^1^. The tree with a good root system tolerates drought and needs watering only when wilting symptoms are notable. Moringa tree is characterized by rapid growth, reaching a height of more than 2 m in less than 2 months and more than 3 m in less than 10 months from planting. The tree may reach a height of between 9 and 12 m within three years^1^. Moringa is a multipurpose tree as it is used for food, fodder, and medicinal purposes^2^. The leaves are good sources of vitamins (A, B, and C), minerals (calcium and iron), and protein^3^.

*Moringa oleifera* grows well in the savannah areas with short grass^4^. It grows under a temperature range of 25–30 °C, but it can survive and grow normally up to 48 °C^2^. It also grows well in areas with an annual rainfall of 1500 mm. *M. oleifera* trees grow at elevations of less than 600 m, but they can grow in areas up to 2200 m above sea level in the tropics^5^. It prefers well-drained sandy loam or clay soil, but it is adversely affected by wet and poorly drained soils^6^. It tolerates soil acidity which ranges between 5 and 9°^1^. Drought-tolerant *M. oleifera* grows well in areas that receive 250–1500 mm annual rainfall and produces much fewer leaves when under water stress^4^. When the annual rainfall is less than 300 mm, the tree needs to have a relatively high water table to be productive^7^. *M. oleifera* is a deciduous tree and most of its leaves are shed during periods of water stress^8^. In dry and semi-arid conditions, it needs watering during the first 2 months, and the tree does not require watering until permanent wilt appears^9^.

*Moringa peregrina* is a medium-sized tree, with a height of 5–15 m^10^. *M. peregrina* usually grows as a deciduous perennial tree, with a thick core, brittle stem, and branches with a bark that is corky in nature. Its stem is erect, branched, and its branches are branched, ascending, or drooping, forming an oval crown or otherwise^11^. *M. peregrina* trees are commonly found in nature as dense shrubs. When planted, it may grow as a very tall tree with a single trunk with few branches^12^. Pruning is usually recommended to enhance branching and pod production and to keep the tree at an acceptable height for pod harvesting^13^. The same author also described *M. peregrina* as a small tree up to 5 m high and has thin branches. The leaves have three leaflets, whole, sharp at the apex, 3–5 cm long, and 2–5 cm wide, but often much smaller and of various shapes. *M. peregrina* grows easily from seeds that take 1–2 weeks to germinate under suitable conditions. *M. peregrina* can also be grown using root tubers, where the tubers are planted vertically with the pointed eyes pointing upwards just below the soil surface^14^. Cuttings can be used successfully for planting *Moringa* species, branches of 1–1.5 m in length are used to make the cuttings. Trees can grow successfully with an annual growth rate that may reach 3–4 m when sufficient ground moisture is available^15^.

Water for irrigation is an important natural resource that supports tree growth and development in the arid and semi-arid tropics^16^. However, there is a growing concern about water availability in arid lands^17^. With the negative effects of climate change, water scarcity will increase in most geographical regions of the world^18^, thus limiting the availability of water supplies, especially in drier areas. Tree water needs to depend on tree species, growth stages, time of year, and prevailing environmental conditions, and therefore, it is necessary to determine water requirement for each type of tree due to the large variation in growth rates^19^. Irrigation for cultivated trees means providing the right amount of water to the plants in the right amount at the right time. Tree growth indicators are generally studied to understand the behavior of trees in light of changing levels of different nutrients or water^20^. The effect of irrigation water quantity and frequency on vegetative growth parameters and chemical composition of *Moringa* trees has been studied^21,22^. The effect of water stress on tree growth and survival has also been extensively studied^23^. It has claimed that in dry and arid climates, *Moringa* can be established using regular irrigation for the first 2 months^1^. Once established, *Moringa* rarely needs watering. The well-rooted tree is usually drought tolerant and needs watering only when wilting is most noticeable. *Moringa* is quite a drought-tolerant but produces much fewer leaves when the tree is under water stress^24^. The needed information regarding the irrigation methods and water requirements and frequencies of *M. oleifera* and *M. peregrina* are scanty, and not much is known about their response to irrigation frequencies. In general, the quantity and quality of irrigation water are very important for trees plantation in arid and semi-arid regions. Frequent irrigation requires careful planning and management to ensure the provision of sufficient water at the proper timing to supplement the growth of these tree species and to obtain high productivity of forage and seeds.

Selenium (Se) is considered a beneficial plant micronutrient and has been shown to enhance plant growth and development as well as plant tolerance to environmental stresses when applied in low concentrations^25^. However, physiological responses of various plant spp. vary significantly to Se application. Se was found in all living organisms and has a significant impact on human, animal, and plant health^26^. The application of Se fertilizer on forage plants has been practiced to build up Se concentration in animals)^27^ and thus become available to humans. Se can be up taken by plants in the form of Selenate (SeO_4_) or Selenite (SeO_3_) or in an organic form^28,29^. The accumulation of Se by plants depends on the form and concentration of the Se as well as the availability of competing ions and the ability of the plant species to uptake and utilize Se^30^. Se foliar application was used to increase the Se content of different plant species^31–34^. Due to its antioxidant properties, Se plays a vital role in promoting plant growth^35^. Adding an appropriate amount of Se can promote plant growth, improve plant quality, enhance plant resistance to water stress, and increase plant yield and Se content^36^. In addition, the use of an appropriate amount of Se can also improve the osmotic-regulating ability of plants and reduce the toxic effects of heavy metals^37^.

To maximize the production value of Moringa species in Saudi Arabia, there is a need to understand the physiological and morphological effect of irrigation frequency and Se fertilizer levels on Moringa species' growth and development. Thus, this study was conducted to determine the effects of irrigation frequency and Se fertilizer levels on the growth performance of *Moringa oleifera* and *Moringa peregrine* under the conditions of the central region of Saudi Arabia by testing the hypothesis that both *Moringa oleifera* and *Moringa peregrina* are significantly affected by the irrigation frequency and Se fertilization, but the response of both species to these inputs varies.

## Materials and methods

All experiments were conducted in Al-Badraniya farm (Al-Ghat district, Saudia Arabia) during the summer season of 2021. The studied treatments included two plant species (*M. oleifera* and *M. peregrina*), four irrigation frequencies (7, 10, 15, and 20 days), and three Se levels (0.0, 12.5 and 25 mg/L foliar spray). The selenium fertilizer treatments were obtained by using organic amino selenium fertilizer 2.5% (Organic Standards Fertlizer Production Company, Riadh, KSA) as Se source, while for the control treatment (0.0 mg Se) only pure water was used. Each replicate was divided into two main plots, which were assigned to the two tree species (*M. oleifera* and *M. peregrina*), and each main plot was divided into four subplots for the four irrigation frequencies, and each subplot was divided into three sub-subplots for the three Se treatments. Each subplot consisted of four 5 m in length furrows. The distance between the furrows is 25 cm and 20 cm between plants within each furrow. A space was left between every two subplots as an implanted space. Also, there was a distance of 2 m between neighboring main plots as buffer zones to prevent the horizontal seepage of water between the different main plots. 100 kg of triple superphosphate and 50 kg of potassium sulfate and 200 kg of urea per ha were added to the soil. The phosphate as well as the potassium sulphate and half of the urea were applied at sowing and the other half of the urea was applied after the first and second plant cuts in equal amounts usually at the peak of the vegetative growth stage.

The experimental treatments (*Moringa* species, irrigation frequencies, and selenium levels) were randomly distributed into the three replicates. The seeds of the two *Moringa* species were planted during the first week of March 2021 and irrigated using drip irrigation till seedling establishment (three weeks) hence the plants were irrigated in accordance with the studied irrigation frequencies. The Se fertilizer treatments were applied as a foliar spray early in the morning every 45 days, and 3 times throughout the experiment.

A sample of five plants was taken randomly from each experimental plot by cutting the selected plants at the height of 10 cm above the soil surface after 100 days from the sowing date as a first cut to determine the vegetative growth (plant height, wet and dry weight of leaves and upper fine stems). The second and third cuts were performed at a 45 days’ intervals from the preceding cut and the same procedures were followed for collecting the needed data each time.

### Statistical analyses

The obtained results were subjected to statistical analyses using the SPSS software program. The data was analyzed to determine the significance of the treatment means and the least significant difference (LSD) was used to compare treatment means at *p* ≤ 0.05.

### Plant guidelines

The plant experiments were in compliance with relevant institutional, national, and international guidelines and legislation.

## Results and discussion

*Moringa oleifera* was noted to have fast seed germination and plant establishment when compared with *M. peregrina* (Visual observation, data not shown). *M. peregrina* had a notable low growth rate under the prevailing environmental conditions in central Saudi Arabia. The differences in growth rate between the two species might be due to their genetic differences. Similar observations were reported where the effects of the environmental conditions on the growth performance of *M. peregrina* was identified^4,38^.

The results of this study include the effect of irrigation frequency (7, 10, 15, and 20 days) and Se fertilizer levels (0.0, 12.5, 25 mg Se/L) on growth characteristics (plant height, leaves, and upper fine stem fresh weight) of the two Moringa species (*M. Oleifera and M. Peregrina*) were discussed as follows.

### Irrigation frequency

The Moringa species plant height, leaves, and stem fresh weight were significantly affected by the studied treatments. The effect of the treatments differed significantly between *M. oleifera* and *M. peregrina*, with *M. oleifera* showing the highest growth performance as compared to *M. peregrina*. The differences between the two Moringa species could be due to their genotypic differences^38^. Irrigated *M. oleifera* plants that received Se were on average taller than those of *M. peregrina* plants. However, the interaction effects of the irrigation frequencies and selenium levels were insignificant. The results showed that the height of *M. oleifera* plants was significantly and positively affected by irrigation frequency as compared to *M. peregrina*.

The drip irrigation frequencies significantly affected the stem height of both *M. oleifera* and *M. peregrina* plants when irrigated every 7, 10, 15, and 20 days (Table 1). However, the two *Moringa* species differed significantly from each other in their plant height response to irrigation frequency (Table 1). The irrigation every 10 days gave consistently the highest plant height of the two *Moringa* species in the three cutting periods, followed by irrigating every 15 days and the least plant height was recorded for *Moringa* species irrigated every week (Table 1). The present study showed that the mean plant height of the two *Moringa* species ranged between 29.8 and 94.3 cm (*M. oleifera*) and 20.9–78.5 cm (*M. peregrina*) in the three cutting periods.

**Table 1:** The effect of irrigation frequency on plant height of *M. oleifera* and *M. peregrina* at different cutting periods.

Treatments	First cut		Second cut		Third cut	
	*M. oleifera*	*M. peregrina*	*M. oleifera*	*M. peregrina*	*M. oleifera*	*M. peregrina*
	Plant height (cm)^1^					
7 days	29.8^c^	20.9^c^	50.2^c^	49.8^c^	74.8^c^	69.9^c^
10 days	34.5^a^	24.8^a^	54.6^a^	59.2^a^	94.3a	78.5^a^
15 days	33.4^a^	24.0^a^	52.3^b^	50.8^b^	80.4^b^	75.7^b^
20 days	30.6^b^	23.1^b^	52.2^b^	50.7^b^	78.5^c^	70.1^c^

^1^Means within each column with the same superscripts were not significantly different using Duncan Multiple Range Test at the 0.05 level of significance.

The two Moringa species recorded the highest plant heights in their third plant cut as compared to the two previous cuts (Table 1). Bruce et al.^39^ claimed that plants when exposed to environmental stress i.e. cutting, it responds very fast when the stress is relieved. This is known as the hardening phenomenon^24^, which was in line with what we obtained in this study where after each cut the plants of the two species showed vigorous growth rates and established themselves very quickly to get the maximum use of the applied irrigation water. Ali et al.^4^ also recorded that irrigation every 10 days showed the highest plant height of the two Moringa species between all irrigation intervals for all the period of the study except the first week, but our data showed the superiority of this treatment in all the period.

The effect of irrigation frequency on leaves' fresh weight of both *M. oleifera* and *M. peregrina* was shown in Table 2. The irrigation frequencies had significant effects on the fresh weight of both *Moringa* species in all cuts (Table 2). Also, the two Moringa species differed significantly from each other in their response to irrigation frequency in leaves' fresh weight (Table 2). The fresh weight of *M. oleifera* leaves had its highest values in the three cuts for the plants that were irrigated every 10 days (Table 2). The highest weights of *M. oleifera* fresh leaves varied between 2.9 and 34.2 g among the three cutting periods. Similarly, *M. peregrina* plants showed significant effects due to irrigation frequency. The fresh weight of the leaves was highest for plants irrigated every 10 days (Table 2). The highest weights of *M. peregrina* fresh leaves varied between 2.7 and 18.9 g in the three cutting periods as affected by irrigation frequencies. The results of the current study were in line with those of Ali et al.^4^, who claimed that irrigation on every 10 days’ basis showed the highest values for leaves weight. In addition, Soliva et al.^40^ indicated that *M. oleifera* plants subjected to mild water stress did recover positively when the water stress was relieved.

**Table 2:** The effect of irrigation frequency on fresh leaves weight of *M. oleifera* and *M. peregrina* at different cutting periods.

Treatments	First cut		Second cut		Third cut	
	*M. oleifera*	*M. peregrina*	*M. oleifera*	*M. peregrina*	*M. oleifera*	*M. peregrina*
	Fresh leaves weight/plant (g)^1^					
7 days	2.2^c^	1.4^c^	9.8^c^	8.7^c^	17.5^c^	14.8^b^
10 days	2.9^a^	2.7^a^	18.4^a^	17.8^a^	34.2^a^	18.9^a^
15 days	2.6^b^	2.6^a^	10.4^b^	14.8^b^	27.7^b^	18.6^a^
20 days	2.2^c^	1.8^b^	10.2^b^	8.9^c^	17.5^c^	15.1^b^

^1^Means within each column with the same superscripts were not significantly different using Duncan Multiple Range Test at the 0.05 level of significance.

The effect of the different irrigation frequencies on the upper fine stem fresh weight of the two *Moringa* species is presented in Table 3. The data presented in Table 3 show that drip irrigation of the plants every 10 days significantly improved both *M. oleifera* and *M. peregrina* vegetative growth performance (in terms of stems' fresh weight) in the three performed cutting periods. The results showed significantly lower values for *Moringa* species stem fresh weight as influenced by the three different irrigation frequencies understudy in the first cut as compared to the other two cuts (Table 3). This might be due to the fact that when plants were cut, severely they respond with very vigorous growth. These results agreed with those recorded by El-sayed and Mahmoud^41^ and Youssef et al.^42^. Also, *M. peregrina* plants had the lowest values (2.3–7.7 g) as compared to *M. oleifera* (3.2–11.3 g) and these differences in stem fresh weight between the two *Moringa* species were significant (Table 3).

**Table 3:** The effect of irrigation frequency on upper fine stem weight of *M. oleifera* and *M. peregrina* at different cutting periods.

Treatments	First cut		Second cut		Third cut	
	*M. oleifera*	*M. peregrina*	*M. oleifera*	*M. peregrina*	*M. oleifera*	*M. peregrina*
	Upper fine stem weight/plant (g)^1^					
7 days	3.2^c^	2.3^b^	4.6^b^	5.0^b^	6.9^c^	5.2^b^
10 days	5.0^a^	3.1^a^	5.5^a^	6.9^a^	11.3^a^	7.7^a^
15 days	4.0^b^	3.0^a^	5.3^a^	6.5^a^	10.7^b^	5.6^b^
20 days	3.9^b^	2.5^b^	4.6^b^	5.0^b^	7.0^c^	5.4^b^

^1^Means within each column with the same superscripts were not significantly different using Duncan Multiple Range Test at the 0.05 level of significance.

### Selenium fertilization

Selenium (Se) is one of the essential micronutrients for human health. Se has an important role in fighting oxidative stress, cancers, bacteria, and viruses^43^. Se deficiency in humans is a potential health risk. This health risk can be checked by the bio fortification of crop plants. For higher plants, Se has also been shown to be essential for crop plants that grow under environmental stresses^44^, but it is not as essential for the normal growth of these plants under normal non-stressed conditions. The bio fortification of forage crops through agricultural practices is possible and through which Se can enter the human food chain via pasture animals and poultry. Se foliar spray and its direct soil application are the two major agricultural Se bio fortification practices^45^. In general, foliar spraying is more effective in increasing Se concentrations in agricultural crop plants, including *Moringa* species^25^. The effects of Se fertilizer foliar application on the growth of *M. oleifera* and *M. peregrina* were studied in this research paper and the findings were discussed below.

The foliar spray of different Se levels had significant impacts on the studied growth characteristics of both *M. oleifera* and *M. peregrina* as obtained in the three cuts (Tables 4, 5 and 6). The different levels of Se considerably enhanced plant height. The maximum plant height was recorded for both *Moringa* species where Se was sprayed at a concentration of 25 mg/L (Table 4). The increase in plant growth parameters due to the application of Se has been recorded by a number of researchers^46–48^. In general, it was observed that the plant height was higher in *M. oleifera* as compared to *M. peregrina* and this trait significantly increased as the Se level increased for both *Moringa* species. However, only slight increases in height were observed in the different cuts with the addition of Se levels to *M. peregrina*, especially at the level of 12.5 mg/L as compared to the control treatment (Table 4). The mean plant height of *M. oleifera* treated with Se at 12.5 and 25 mg/L concentration was 2.38%, 3.05%, and 0.62% higher than that of the control plants in the three cutting periods, respectively. While the mean plant height of *M. peregrina* treated with Se at 12.5 and 25 mg/L concentration was 5.24%, 3.51%, and 6.41% higher than that of the control plants in the three cutting periods, respectively. However, no significant increase in *M. peregrina* plant height was observed when Se was applied at the rate of 12.5 mg/L as compared to the control in the 1st cut, and the same was true for *M. oleifera* plant height when Se was applied at a rate of 12.5 mg/L as compared to the control treatment in the 3rd cut (Table 4). Also, no significant difference was recorded for *M. peregrina* plant height when Se was applied at a rate of 12.5 mg/L as compared to that applied at a rate of 25 mg/L in the 2nd cut (Table 4). Earlier studies indicated that Se at low levels enhances plant growth^49,50^. However, higher Se levels reduced plant growth^51^.

**Table 4:** The effect of selenium fertilization (mg Se/L) on plant height of *M. oleifera* and *M. peregrina* at different cutting periods.

Treatments	First cut		Second cut		Third cut	
	*M. oleifera*	*M. peregrina*	*M. oleifera*	*M. peregrina*	*M. oleifera*	*M. peregrina*
	Plant height (cm)^1^					
0.0	42.0^c^	21.5^b^	55.8^c^	51.3^b^	80.7^b^	68.6^c^
12.5	43.0^b^	22.7^b^	57.5^b^	53.1^a^	81.2^b^	73.0^b^
25.0	44.2^a^	25.5^a^	60.0^a^	53.5^a^	84.2^a^	76.05^a^

^1^Means within each column with the same superscripts were not significantly different using Duncan Multiple Range Test at the 0.05 level of significance.

**Table 5:** The effect of selenium fertilization (mg Se/L) on fresh leaves weight of *M. oleifera* and *M. peregrina* at different cutting periods.

Treatments	First cut		Second cut		Third cut	
	*M. oleifera*	*M. peregrina*	*M. oleifera*	*M. peregrina*	*M. oleifera*	*M. peregrina*
	Fresh leaves weight/plant (g)^1^					
0.0	2.3^b^	1.5^c^	4.6^c^	4.5^c^	6.0^c^	6.8^c^
12.5	2.4^b^	2.0^b^	5.0^b^	5.1^b^	8.2^b^	7.6^b^
25.0	2.6^a^	2.9^a^	5.3^a^	7.7^a^	13.8^a^	8.0^a^

^1^Means within each column with the same superscripts were not significantly different using Duncan Multiple Range Test at the 0.05 level of significance.

**Table 6:** The effect of selenium fertilization (mg Se/L) on fresh shoots weight of *M. oleifera* and *M. peregrina* at different cutting periods.

Treatments	First cut		Second cut		Third cut	
	*M. oleifera*	*M. peregrina*	*M. oleifera*	*M. peregrina*	*M. oleifera*	*M. peregrina*
	Upper fine stem weight /plant (g)^1^					
0.0	2.3^b^	1.5^c^	4.6^c^	4.5^c^	6.0^c^	6.8^c^
12.5	2.4^b^	2.0^b^	5.0^b^	5.1^b^	8.2^b^	7.6^b^
25.0	2.6^a^	2.9^a^	5.3^a^	7.7^a^	13.8^a^	8.0^a^

^1^Means within each column with the same superscripts were not significantly different using Duncan Multiple Range Test at the 0.05 level of significance.

The results of the current study indicated the beneficial effects of the foliar application of Se on the *Moringa* species growth when the element was sprayed at the peak of the vegetative growth which indicated the importance the foliar timing whether during the day (early in the morning) or the stage of plant growth (during the excessive vegetative plant growth). The low Se levels such as those used in this study caused an increase in photosynthetic rate and chlorophyll content and thus enhanced shoot growth and the reverse is true for high Se concentrations^51^. The various levels of Se studied improved the leaf fresh weight of both *M. oleifera* and *M. peregrina* during the three cutting periods, and there was a significant difference in the responses of leaf fresh weight of the two Moringa species to the application of the different levels of Se (Table 5). In the first cut, *M. oleifera* plant receiving the 12.5 mg/L Se did not differ significantly in fresh leaves weight as compared to the control (Table 5), while the high level of Se (25 mg/L) had the highest leaf fresh weight for *M. peregrina* in the 1st cut which was significantly different from both the 12.5 mg/L and the control treatments, and was also higher than that of *M. oleifera* (Table 5). In the 3rd cutting period, the maximum increase in the leaf fresh weight of both *M. oleifera* and *M. peregrina* plants was observed when Se fertilizer was applied. The lowest leaves fresh weights were recorded at the control treatments, followed by those receiving 12.5 mg/L Se and then those receiving 25 mg/L Se (Table 5).

The data presented in Table 6 show that there were significant responses to the addition of Se fertilizer on the upper fine stem fresh weight of both *M. oleifera* and *M. peregrina* as compared to the control. However, Se application at the rate of 25 mg/L to *M. oleifera* was superior to the other two treatments (Table 6). In general, the response of *M. peregrina* was lower as compared to that of *M. oleifera* to Se fertilization on the upper fine stem fresh weight (Table 6) in the three cuts. It is clear from the published data and the data recorded in this investigation that the impacts of Se application on crop plants' performance depend on its level in the growth media and the applied Se levels.

## Conclusions

The determination of the irrigation requirements and irrigation frequencies of *Moringa* species in the Kingdom of Saudi Arabia is important for irrigation water management due to irrigation water scarcity in the country. This research investigation showed significant differences between the two *Moringa* species (*M. oleifera* and *M. peregrina*) in response to drip irrigation frequency and Se fertilizer application. The research study concluded that both *Moringa* species when irrigated using a 10 days’ drip irrigation frequency had high plant growth performance, especially *M. oleifera* which could be recommended for cropping in central Saudi Arabia. The experiment also indicated that the foliar application of 12.5 mg and 25 mg Se/L water spray during the vegetative stage significantly increased growth performance. The Se impact on *M. oleifera* and *M. peregrina* growth depends on its concentration. The *Moringa* species growth parameters were enhanced by the 12.5 mg and 25 mg/L Se treatments as compared to the control (0.0 mg Se/L). The experiment also confirmed that foliar application at the vigorous vegetative growth stage could be considered the most effective methodology for Se enrichment in *M. oleifera* and *M. peregrina* plants.

## Data Availability

The data sets used and/or analyzed during the current study available from the corresponding author on reasonable request.
